# Application of Analytical Methods for the Comprehensive Analysis of Oleogels—A Review

**DOI:** 10.3390/polym13121934

**Published:** 2021-06-10

**Authors:** Andreea Pușcaș, Vlad Mureșan, Sevastița Muste

**Affiliations:** Department of Food Engineering, Faculty of Food Science and Technology, University of Agricultural Sciences and Veterinary Medicine Cluj-Napoca, 3-5 Manăștur st., 400372 Cluj-Napoca, Romania; andreea.puscas@usamvcluj.ro (A.P.); sevastita.muste@usamvcluj.ro (S.M.)

**Keywords:** oleogel properties, edible organogel, oil structuring, structured lipids characterization

## Abstract

Numerous empirical studies have already been conducted on the innovative fat-replacing system defined as oleogel, creating a real urge for setting up a framework for future research, rather than conducting studies with arbitrary methods. This study re-evaluates the utility of some analyses and states some conclusions in order to eliminate the reluctance of food processors and consumers towards the utilization of oleogels as ingredients. The review presents extensively the methods applied for the characterization of various oleogels, while highlighting their addressability or inconveniences. The discussed methods were documented from the research published in the last five years. A classification of the methods is proposed based on their aims or the utility of the results, which either describe the nano-structure and the network formation, the quality of the resulting oleogel or its suitability as food ingredient or other edible purposes. The general conclusions drawn for some classes of oleogels were also revisited, in order to ease the understanding of the oleogel behaviour, to encourage innovative research approaches and to stimulate the progress in the state of art of knowledge.

## 1. Introduction

An oleogel can be regarded as a substantially diluted system, which despite its high liquid content, displays solid-like properties and, due to some molecules with gelation capacity, a network is formed where the surface or capillary forces immobilize vegetable oils or mixtures of fatty acids into non-flowing conditions [1,2]. Organogelators are classified as having a low molecular weight with less than 3000 Da, or as high molecular weight gelators [3]. According to their chemical characteristics, gelators are lipid and non-lipid compounds or inorganic particles. Most of the low-molecular weight gelators are lipid in nature and present amphiphilicity, whereas the rest of them, polymeric organogelators, cellulose derivatives, proteins, water-soluble polysaccharides and inorganic particles, are non-lipid-based gelators [1,4]. The low volume of low-molecular weight organogelators allow more space for hydrogen bonding and strong networks will be developed even when they are used in low concentrations. According to the principles of the structuring mechanism, organogelators can be crystalline particles or self-assembled structures of low molecular weight compounds, which are used in the direct methods of oleogelation. In the indirect methods of the oleogelation, self-assembled structures of polymers or polymeric strands can be involved, and the structuring mechanism is based on either developing foams, miscellaneous structures, emulsions, solvent exchange or aerogel templating [5,6].

Numerous oleogels were proposed as ingredients in food prototypes, but the functionality provided by fats (texture, mouthfeel, structure, gluten coverage) was not fully achieved and the up-scaling of the oleogel formation or the industrial application as an ingredient have not been reported yet. A very recent review address the oleogel use and estimated production volumes and prices for selected oil structuring agents [7]. It stated that some waxes are byproducts and are economically feasible and it presents their potential production volumes and prices from different producers. Furthermore, the amount of Oryzanol produced from rice and the price per oleogel is estimated. Moreover, paper waste and food waste are a good source for the production of cellulosic derivates so, their availability is also high, while the price range is diverse, methylcellulose being the most accessible. Mono- and diglycerides of fatty acids can be produced in large quantities, but their usage is not different from the triacylglycerol (TAG) structuring method, from a nutritional point of view. For this reason, it is necessary to establish which oleogel system is feasible as an ingredient, which of its property is relevant for what application, and also to apply other methods or analysis which will eliminate the reluctance of food processors and consumers towards the utilization of oleogels as functional ingredients.

For the characterization of oleogels, we propose in current manuscript that the comprehensive analysis of oleogels and their gelation mechanism should be analyzed on three different levels, from nanometre to the microscopic and macroscopic dimensions. The primary structures (angstrom to nanometre scale) and the bonds occurring between the atoms can be determined on a nanometric scale, because their geometric parameters such as surface area and volume, are directly affected by cooling rate and shearing during gelation [8]. This domain on a nanometric scale can be examined more in-depth by Fourier-transform infrared spectroscopy (FTIR) measurements or Raman Spectroscopy or by microscopy, the crystal’s size measurements being also possible in the case of crystalline gelators, with the use of X-ray diffraction (XRD) analysis.

The secondary structure, which reflects the transition from a nanometric to a micrometre scale, is represented by the morphology of the aggregates, such as micelles, vesicles, fibbers, ribbons or sheets, depending on the nature of the organogelators and is based on describing the underlaying mechanism of the formation of the microstructure.

The resulting gel is visible up to a millimetric scale and refers to the complexity of the networks, which either entraps liquid oil, or precipitates the gelator from the solution [2], the physico-chemical and sensorial properties of the resulting oleogel being of high interest in this domain.

The process of oleogelation requires the application of temperature treatments over a mixture of structuring substances and vegetable oils, with or without the application of shearing forces. For this reason, thermal analysis, rheology and optical detection methods are the fundamental methods of analysis applied to characterize the formation of an oleogel. Through these means, the melting and crystallization temperatures or enthalpies, critical gelation concentrations, the linear viscous regions, or the microstructure of the resulting structure can be determined.

A very recent review discusses critically the experimental strategies and the main determinations used in the characterization of the oleogels, such as the critical concentration, solubility and crystallization, the sol gel transition assessed by rheology and DSC, hardness and deformation, microscopy and oil binding capacity [9].

The quality of the resulting oleogel is given by the oxidation state or the oil binding capacity. The oxidative state of an oleogel will influence the nutritional quality and the storage stability. For this reason, analytical determination, such as the peroxide values (PVs), p-anisidine (p-AV), and thiobarbituric acid reactive substances (TBARS numbers), are often determined, along with accelerated oxidation tests. The oil binding capacity is measured with centrifugal or dispersion analysis and it is influenced by the interactions occurring in the oleogel.

Textural parameters, the solid fat content, colorimetry and sensorial analysis will offer information about the functionalities of an oleogel and their applicability as fat replacing systems in food products.

To sum up, the reviews and research articles published so far, related to edible oleogels, in our opinion, either explore different structuring agents or propose the usage of different vegetable oils with positive implications on the health of consumers, describe the structuring mechanism and evaluate the quality of the resulting oleogels, or asses their functionality as ingredients in food products, by comparing them with some conventional fat systems. Based on the critical discussion of the methods used so far for the comprehensive analysis of oleogels and the results obtained for different systems, it is questionable as to whether an analysis is easing the understanding of the oleogels behavior. It is worth conducting and developing more innovative research approaches in order to stimulate progress in gathering knowledge through state-of-art methods.

**Figure 1:**
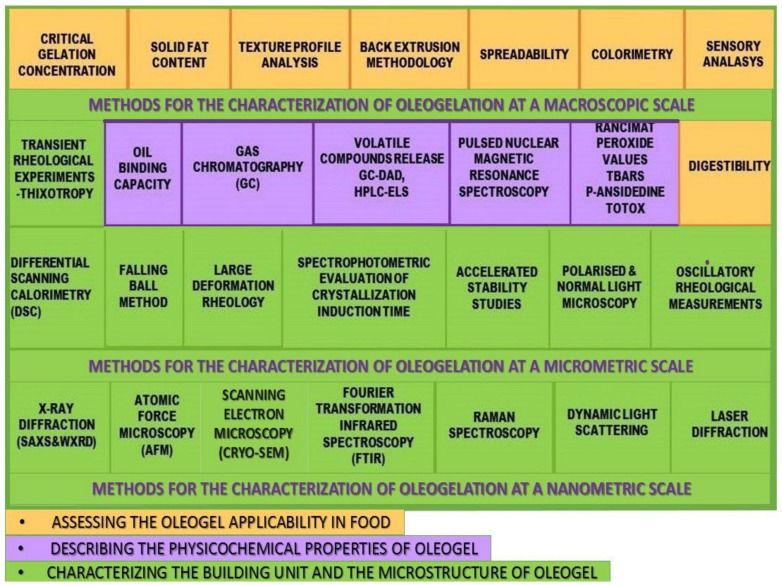
A proposed overview classification of the analytical methods of oleogel characterization.

The current review proposes a classification overview (Figure 1) of the methods used for evaluating the edible oleogels, based on their purpose: methods which are describing the structural units, the gel’s network development or its response towards external factors, methods which assess physicochemical properties or the functionality of the oleogels as a fat replacing system.

The usage of oleogel as an ingredient is now regarded also as a complementary path for different targets including the nutritional improvement due to the polyunsaturated fatty acids delivered by the oil and for the bioactivity of the gelling agents [10]. The World Health Organization (WHO) states that if the consumption of trans fatty acids and saturated fatty acids is replaced with polyunsaturated fatty acids, a decrease in the risk of coronary heart disease occurs, and also calls for research to develop methods of analysis for assessing fatty acid intakes in individuals, including development of robust biomarkers [11].

Oleogels also demonstrate techno-functionality in food products where oil migration, syneresis, or fat blooming are among the major defects, due to their properties such as oil binding capacity. Moreover, they are a promising mechanism for the delivery of lipophilic substances, improving the bio-accessibility of several compounds, while the emulsionated oleogels are explored as carriers of lipophobic substances. Their incorporation in food meets some challenges, such as the formulation adaptability, costs and revenue, but also the regulations and approvals regarding food ingredients. There are also some opportunities, which will hurry the transition of oleogels from the research laboratories to the consumers menus, such as the promising results of academic research, the strong collaboration between the academic and industrial sectors, the need to use more environmental friendly ingredients, and a widespread marketability [10].

It has been reported that the ability of a gelator molecule to gel a solvent is influenced by a balance between the solubility and insolubility of the gelator within the solvent [12,13]. Oleogel properties are influenced by intrinsic factors such as the type of oil and the oleogelators involved in the process [14,15], the interactions amongst the gelator and the oil molecules, but also by impurities or minor components [15]. The influence of the type of oil is explained as follows: the presence of a greater unsaturation degree and bended chains cause a larger molar volume and a higher conformational freedom, and affect the structuring mechanism, different behaviours being registered for polymeric strands in comparison to the lamellar phases [16]. Solvent properties and solubility parameters influence numerous self-assembly gelators, along with external conditions affecting primary structures and forces which lead to epitaxial growth into axially symmetric elongated aggregates [15,17]. Moreover, external factors such as cooling rate, application of shear or the addition of surfactants, can be controlled in order to tailor edible oleogels suitable for the replacement of saturated or trans fats in foods [18]. High molecular weight gelators present predominantly hydrophilic characteristics and insolubility in non-polar solvents and indirect methods of oleogelation are necessary to be addressed, except for ethyl cellulose and chitin, which are suitable also for direct dispersion [1,19].

Proteins can be used for oleogelation, the formation of an oil-in-water emulsion being necessary, along with the addition of polysaccharides and the drying of the system, which results in the formation of a polymeric network filled with oil and a high internal phase emulsion (HIPE). The formation of a gel using self-assembled structures of polymers or polymeric strands can be achieved due to the formation of capillary suspensions at the addition of a secondary fluid immiscible in the primary solvent [20]. When proteins are used as gelators for oil, and water is added to the system, it can act as a physical bridge between protein aggregates; capillary suspension phenomena are also observed when silica particles are used for structuring purposes [19].

In non-polar solvents, interactions between colloidal particles are mainly governed by steric interactions and hydrogen bonding, for silica particles, gelation being achieved when the solvent is incapable of forming hydrogen bonds with the hydroxyl groups on the surface of the silica particles [20]. Recently, the surface chemistry of fumed silica particle manipulation was studied in order to optimize its structuring activity. When silica powders are wetted by edible oils, the silanol groups on the particle surfaces are sites for molecular adsorption of species capable of forming hydrogen bonds, or of undergoing donor–acceptor interactions, the gelation capacity of this powders being in correlation with the number and distribution of silanol groups [21].

Polymer–polymer hydrogen intermolecular bonding is the reason for the ability of ethyl cellulose (EC), a linear polysaccharide derived from cellulose, to structure oils. This oleogelator consists of a cellulose backbone with the replacement of some of the hydrogen of the cellulose’s hydroxyl end groups with ethyl end groups, different degrees of substitution being possible [22]. For oleogels structured with ethyl cellulose, the formation is based on heating the system above the glass temperature of ethyl cellulose, followed by cold-induced gelation, the temperature of gelation varying with the molecular mass of the ethyl cellulose, but also with the nature of the oil. Antioxidants might be also added to the formulation, because glass transition temperature of the ethyl cellulose is around 140 °C and causes thermal oxidation. Gelation temperature and also critical gelation concentration were reduced by using a mixture of the lauric acid and ethyl cellulose as gelators [23]. This can be due to the carboxyl head group of the lauric acid and free hydroxyl of ethyl ceullulose, causing polymer–polymer interactions through possible van der Waals interactions, but also due to polymer–solvent interactions between lauric acid attached to EC and the tri-acylglycerols of the canola oil [23]. Setting the temperature is also important for the quality of this oleogels, as higher temperatures allow a slower, more ordered cross-linking process, which produces a stronger network, in comparison to quick cooling and faster interactions between polymer chains and less ordered hydrogen bonds [24]. Cellulosic structurants both synthetic and natural, are also proposed as oil structuring agents, due to their hydrogen bonding ability, amphiphilicity and several hydroxyl groups in the composition, the hydrophobic chain stabilised oleogels demonstrating potential as ingredients in meat products [25,26], pastry [27,28] and confectionery [29].

## 2. The Nanometric Scale Characterization of Oleogels

### 2.1. Oleogel’s Building Units

In the case of crystalline oleogels, crystallization of lipidic structures occurs, mostly due to the assembly of triacylglycerol molecules into nano-platelets at the nanoscale level [12]. Furthermore, depending on the structuring agents or external conditions (cooling rate and shearing during gelation), crystals might suffer polymorphic transitions [4].

X-ray diffraction (XRD) is a technique providing information about the crystalline nature of the gelling molecules, the shape they develop, the mean size, the way they arrange themselves in the oil and their polymorphic form. The morphology of these crystals can be studied by using the small-angle X-ray scattering (SAXS) technique. The Bragg peaks position is commonly used to determine the longitudinal molecular packing arrangement in comparison to the (001) d-spacing of consecutive atomic crystallographic planes [30].

Using synchrotron light X-ray diffraction, microcrystals of monoglycerides were revealed in the structure of an oleogel developed under ultrasonic standing waves, while in oleogels which crystallized in statical conditions, the microcrystals were absent. The monoglycerides in the sample treated with ultrasounds of 1 MHz, exhibited the triclinic β-form, and a lamellar thickness of 48 Å–49 Å was observed, while for the rest of the samples the thermodynamically stable form was observed, namely the hexagonal α-form with a slightly bigger lamellar thickness dimension [31].

The usual information reported is the long and short spacing of crystals, which define a particular polymorphic form, yet X-ray diffraction patterns can also provide additional and valuable information on the structure of a fat crystal network. Depending on the d spacing values from the XRD study, one can suggest a probable molecular packing arrangement in the self-assembled gel state [32]. Wax-based oleogels display similar XRD patterns of orthorhombic perpendicular subcells and β′ polymorphic forms; for this reason they are highly suitable for the replacement of those fats which offer plasticity and mouthfeel to the food product [33,34]. An exception is reported for beeswax oleogel, which exhibited in the small diffraction angles only one peak at a low d-value (0.415 nm), which is a characteristic of a hexagonal symmetry [35]. Beeswax arranged itself in different manners in the medium chain triglycerides (MCT) and long chain triglycerides (LCT), depending also on its concentration, different spacing and placement between crystals, and a lamellar conformation being denoted by the distance between peaks, which was in the 1:2:3:4 relation [36].

Understanding the phenomenon of polymorphism is essential in designing new types of fats, given the ability of a chemical compound to form different crystal structures, the polymorphism being influenced by the type of fatty acids in the structure of the fat. Crystallized fat can be found in the following forms: α and γ—not at all stable, with a hexagonal assembly of triglyceride chains, which at heat treatment will turn into β′ (meta-stable) or β (stable). The β′ polymorphic shape of fat crystals has an intermediate stability, and a perpendicular orthorhombic arrangement and is suitable for margarine and shortening products, with an optimal morphology and microstructure, which determines the textural and rheological parameters of the product. The polymorphic form β has a higher stability and parallel triclinic packaging and is found in chocolate products containing cocoa butter. The melting point of vegetable oil is influenced by the polymorphic form of the component triacylglycerols, increasing with the increasing crystal stability and a difference in molecular density due to different assembly [37].

In a study where oleogels were proposed as alternatives for cocoa butter, corn oil structured with 10% monoglyceric stearate exhibited diffraction peaks which can be correlated to both β′ and β polymorphs, while corn oil structured with a mixture of β-sitosterol and lecithin, exhibited only β polymorphs and ethyl cellulose oleogel did not exhibit diffraction peaks [29].

Monoglycerides from hydrogenated palm oil (MGP) was used to structure refined sunflower oil form bi-layers, the distance between the layers being determined in the small angle region, while in the wide-angle region they exhibited a peak which denotes a β-polymorphic form and an in-plane ordering of triclinic configuration [38]. The phytosterol (PS) crystals present in the refined sunflower oil oleogel were reported as forming a pseudo bilayer structure of repeating units, mainly composed by sterane cores, appearing in more forms such as anhydrate, hemihydrate and monohydrate, exhibiting multiple peaks in the wide-angle region during XRD analysis [39]. The wide-angle X-ray diffraction (WXRD) exhibited by a pure substance is like a finger print of that substance [40], but phytosterols like γ-oryzanol are usually made up of more substances depending on their origin [41]. When MGP and PS were used as gelators in a ratio of 8:2, it resulted in the formation of a novel peak during the XRD analysis, which might be due to a complex formation or due to the crystal modifier habit of MGP [38]. XRD is a useful tool in order to examine the co-crystallization or the formation of mixed crystal systems when structuring with multiple gelators. Stearyl alcohol:stearic acid (SO:SA) mixtures used for structuring edible oils led to the formation of new crystals and co-crystallization for the intermediate ratio mixtures where stearic acid was predominant, as observed in SAXS and WAXS regions [30]. Mixed crystals were not revealed in the study of binary blends of waxes (rice bran wax, sunflower wax and berry wax) used for preparing an oleogel from rice bran oil [42]. Both the β′ polymorph and γ polymorph were observed in the oleogels formed with RBX:berry wax mixture and SFW:berry wax [42].

The influence of Tween 20 on the formation of stearic acid molecular layers and the modification of crystal size in a soybean oleogel was revealed by an XRD study, beside the polymorphic state of stearic acid revealed by the short spacing and the order and thickness of the molecular layers by the long spacing peaks [43]. Stearic acid presents as a single gelator for oleogels, and is displayed in one molecular packing arrangement, namely a double layer structure, and crystallized into the most stable polymorphic form, the C form, among the four different polymorphic forms are reported for stearic acid: A, B, C, and E [30,44].

XRD diffraction patterns of the sunflower oil oleogel developed with stearic acid and β-sitosterol used as single or mixed oleogelators displayed long spacing peaks of high intensity, except for the oleogel structured with solely stearic acid [45]. When the oleogel was developed with almost equal molecular ratios of the structuring agents, short spacing peaks were of a high intensity, different polymorphic transitions being revealed for different combinations [45].

The most efficient crystallization temperature required for the formation of a well-ordered structure was assessed using the XRD profiles at the wide-angle region of an oleogel formed by the indirect method of emulsification, concluding that for a corn oil oleogel crystallized at 0 °C, better structural parameters and crystallinity is achieved than for those crystallized at −18 °C, 5 °C and 25 °C [46].

Microscopy allows the visualisation of different molecular oleogelators, the underlying mechanisms of the formation of the oleogel due to a single or multi-component structuring system and some possible interactions. Different techniques allow the visualisation of different depths and resolutions, some of them requiring a prior preparation of the analyzed sample and special equipment. Cryogenic-temperature transmission electron microscopy (cryo-TEM) allows the analysis of the morphology of the basic building blocks, the dimension of the observed platelets varying with the supersaturation of the oleogel [47]. When the initial stage of physical gelation is the self-assembly or self-aggregation of the gelator molecules and the process is influenced by the surface area of the gelator or surface roughness, a scanning electron microscope can also be used for analysis [16]. The major inconveniences related to electron microscopy are the necessities of removing the liquid phase from the system with organic solvents, which might affect the structure or cause polymorphic transitions.

**Figure 2:**
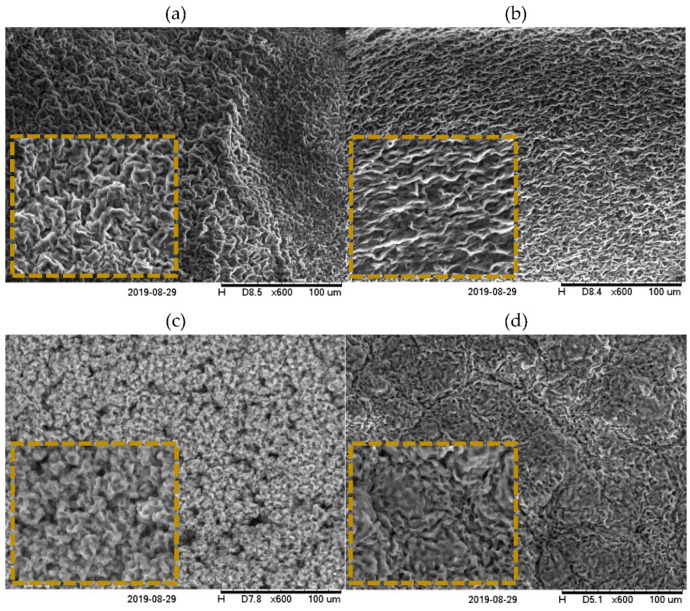
Scanning electronic microscopy (SEM) images of Beeswax olgeogel (**a**), 25:75 mixture of glycerol monostearate and beeswax (**b**), 25:75 mixture of glycerol monostearate and sunflower wax (**c**), and 50:50 mixture of glycerol monostearate and sunflower wax (**d**) oleogels. Magnification of 600×. Scale equivalent to 100 μm. The inset figures correspond to 1000× magnification. Taken from Barroso et al. [16]. Copyright 2020 Creative Common Attribution License.

Scanning electron microscopy (SEM) and atomic force microscopy (AFM) revealed that the morphologic units of the crystal unit of waxes, which initially were considered to be “needle-like”, are platelets [48,49,50], as shown in Figure 2a. Since they are having the same morphology, oil binding capacity of the waxes is considered to be influenced by the surface area, surface roughness and surface quality (chemical nature) and surface engineering strategies are needed for controlling the oleogel functionalities. Crystal size of sunflower wax is increased with wax concentration, mostly because of available crystalline material, making further crystal growth possible [51]. Despite this, on a molecular level, waxes appear to be different, depending on the chemical composition and packing of the carbon atoms [7].

Monoglicerydes were reported to change the configuration of the beeswax network to a structure reassembling platelets piled upon each other (Figure 2b). Moreover, when glycerol monostearate (GMS) was mixed with sunflower wax (SFW), different crystal networks were observed: a sponge-like network for the GMS:SFW (25:75) combination and a platelet organization with a different display for GMS:SFW (50:50) [16].

Atomic force microscopy was used in exploring an oleogel containing 10% or 20% mixture of γ-oryzanol and β-sitosterol, and it was revealed that fibrils present numerous junction zones of different aspects (X, Y, 4 point junction) and their interaction was a key feature in the type of the structure, stacking being due to the hydroxyl and methoxyl groups of the ferulic acid contained by the γ-oryzanol [52].

### 2.2. Structurants, Oil, and Minor Compounds Molecular Interaction

During the oleogel formation, individual aggregates are interacting and, based on non-covalent interactions such as hydrogen bonds, Van der Waals interactions, π stacking or metal coordination, are forming supramolecular structures [3] such as tapes, rods, fibbers and sheets, which entrap the liquid vegetable oil [14]. The study of the intermolecular interactions occurring among the molecules of the structuring agents or between them and the vegetable oil can be performed by Fourier Transformation Infrared Spectroscopy (FTIR) and Raman Spectroscopy. The presence of some bio-active compounds such as phenolic compounds or carotenoids, provided by the oil, can also be detected. These are quick and non-invasive methods of analysis which were successfully applied on different food products, including the characterization of edible oils and oleogels. The oleogel structural configuration can be assessed by the changes in the shape and frequency of the bands specifically corresponding to molecular vibrations, namely specific chemical bonds generating specific Raman shifts, while in case of FTIR, specific absorbance can be read in specific wave-length regions.

Raman spectroscopy was used to analyze an oleogel formed with a mixture of berry wax and lecithin; the oleogel structured with this mixture exhibited peaks similar to the oleogel formed with solely berry wax, except for a peak at 720 cm^−1^, which can be related to the C–N stretching of the PO_2_^−^ from the composition of the lecithin [53]. Moreover, the entropic hydrophobic effect was detected, which depicts a limited interaction between the hydrophilic head groups and the solvent [53].

Raman Microscopy coupled with an FTIR spectrometer were used to reveal the intermolecular hydrogen bonding between sitosterol and oryzanol and to eliminate the fluorescence effect; the samples were placed in the beam path for ~20 min prior to measurement to photo-bleach chromophores [41]. The aromatic ferulate group contained by oryzanol, which leads to π–π interactions with other phytosterols, and can be visualized at 1183 cm^−1^ where peaks are assigned to the methoxy-group and at 1583 cm^−1^ due to the aromatic ring [41]. A peak at 3441 cm^−1^ reveals the intermolecular hydrogen bonds between oryzanol and sitosterol.

In case of the oleogels structured with ethyl cellulose, a weak band at around 3473 cm^−1^ was noticed and depicts the OH stretching vibrations and the intramolecular or intermolecular hydrogen bonding between polysaccharides [26]. The FTIR analysis of soybean oil structured with different mixtures of cellulosic derivates such as Avicel^®^, carboxymethylcellulose, ethyl cellulose, or α-cellulose, revealed peaks above 3000 cm^−1^ specific to the different interactions occurring between their functional groups [54]. The study of an oleogel developed with Span 60 dissolved in mustard oil using FTIR, resulting in the formation of a peak around 1750 cm^−1^ and illustrating the CO from the composition of Span 60 and a peak around 3500 cm^−1^ due to its hydroxyl groups [55]. Propolis wax was used to structure different oils; the FTIR analysis showed a peak around 2900 cm^−1^ due to the long chain alkanes from its composition [56]. β-sitosterol was examined with FTIR and a broad peak was observed at 3421 cm^−1^, but after its inclusion in sunflower oil with or without being combined with stearic acid, the peak was strongly decreased and shifted, indicating less intense hydrogen bonding and more complex interactions [45]. An interesting peak in the 1700 cm^−1^ region, which is not discussed in the study of Yang et al. [45], is that of the oleogel sample developed with only β-sitosterol. The peak is exhibited with a lower intensity in the oil sample due to the lipidic acyl chains and is absent in the sitosterol sample, revealing some interactions between the sunflower oil and the hydroxyl groups of the sterol [45]. Moreover, the FTIR measurements revealed that the particle sizes of the structuring agents influence the degree of hydrogen bond formation in oleogels from whey protein aerogel particles [57].

One major drawback of FTIR is the overlapping of peaks and the mixed signals due to the complex chemical composition and similar constituents. The functional groups contained in the rice bran wax were reported to overlap with those of the protein structures, and thus it would be hard to examine protein-based foods containing wax oleogels [58]. Bioactive compounds such as carotenoid might also be hard to examine by FTIR means, since OH functional groups such as the waxes from the composition of the oleogel or oxidation by-products form peaks in the same regions. For instance, an oleogel developed with beeswax and different oil phases exhibited a broadened and more intense peak in the 1746 cm^−1^ region than the oil or the wax due to oxidation [36].

Dynamic light scattering can be involved in the study of the aggregation process of the structuring agents. In the study of [36], the light intensity as a function of temperature is monitored and the results highlighted that beeswax crystallization behavior is influenced by its concentration in a specific temperature range, or by the oil type. Laser diffraction was also involved in tracking the particle size changes of an oleogel during storage, in order to evaluate the aggregation of the particles [59].

## 3. Evaluation of Oleogels in the Micrometric Domain

### 3.1. Phase Transitions

Oleogelation conducted with crystalline structures such as waxes, monoglycerides, phytosterols, long-chain fatty acids and/or mono-hydroxyl alcohols, etc., can be characterized by Flöter et al. [9] The melting peak broadness (from T_onset_ to peak point) offers information about the complex composition and thermo-responsive behavior of an oleogel, which becomes more fluid-like after heating. A simple method to determine the *T*_m_ (melting point) of the organogels is by the falling ball method. When the ball attached with a melting point apparatus starts to move into an oleogel during heating, the gel-sol transition temperature can be registered [60].

Moreover, melting and crystallization enthalpies determined from the area of the endothermic and exothermic peaks, respectively, could characterize the oleogelation from a calorimetric point of view. Since entropy indicates the degree of disorderness, lower entropy values are usually associated with the thermally stable organogels. The ratio between the enthalpy value of the neat gelator (ΔH_n_) and the melting enthalpy in oil (ΔH_o_) results in the crystallization percentage of the gelator in oil [61].

The type of the oil influences crystallization, saturated oils leading to DSC melting and cooling profiles at higher temperatures [46]. The concentration of the structuring agent [55] or the ratio between the structuring agents from a mixed component [38] leads to different thermal stability and different thermograms. For example, a higher concentration of SPAN 60 or, for another oleogel, a 6:0 mixture of monoglycerides—phytosterols in comparison to the 6:4 mixture—were showing faster crystallization temperatures. Furthermore, it is already well stated that the onset melting temperatures and melting points of wax-based oleogels are lowered in comparison to the ones of the corresponding wax analyzed solely as substances. Moreover, for an oleogel formed with medium- or long-chain triglycerides and different beeswax concentrations, increased crystallization temperatures indicate the development of a strong crystal network [36].

The gelling points can be determined with a rheological measurement and a starch pasting cell, where an oleogel is placed and subjected to a decreasing thermal treatment or to isothermal treatment, in order to further examine the gelling properties [42]. Large deformation rheology (flow measurements) can be conducted in order to assess the crystallization behavior of an oleogel. The gelling temperature (T_gel_) is the temperature corresponding to the cross point between the elastic and plastic modulus (G′ = G″); the dynamic yield stress (τdy) value is also of high interest to evaluate whether oleogel is more liquid-like [62]. The flow index (*n*) can be determined using the Hershel–Bulkley model, which will indicate whether an oleogel is presenting shear thinning (*n* *<* 1) or shear thickening (*n* > 1) behavior. The Arrhenius Equation describes the non-linear inverse relationship between viscosity and temperature and can be used to determine the activation energy (Eα) used to describe the influence of temperature changes over the oleogels [63].

The crystallization induction time can be evaluated with a spectrophotometer equipped with a cell holder, which thermoelectrically controls temperature, and the analysis is based on registering the changes in absorbance at 600 nm over time, the crystallization induction time being considered the onset of the turbidity development calculated when the absorbance deviates from the baseline by 1% [61]. The crystallization kinetics of the oleogels can be also studied with an optical density device, coupled with a temperature-controlling system, both in transmittance or absorbance mode [43].

Accelerated stability studies can be conducted by thermo cycling or freeze–thaw cycling, and syneresis measurements to assess the change in gelation time with consecutive freeze–thaw cycles. A thermo cycling or freeze–thaw cycling method involves incubation of the freshly prepared oleogels at 65 °C for 15 min until they are a liquid, followed by gelation when the time was noted and another incubation at 4 °C for 15 min after which it is stored at 25 °C for 48 h. The cycle is repeated for 6 cycles. Soybean and olive oil-based oleogels structured with SPAN 40 or SPAN 40 and TWEEN 80 mixture were found to be stable up to 6 thermo-cycles [64], but an increase in the gelation time was observed after each cycle, for the olive oil oleogels structured with bot SPAN 40 or the mixture.

These studies are highly relevant for the practical application of oleogels, in order to evaluate whether they can withstand a wide range of temperature conditions during their shelf-life or transportation.

### 3.2. Characterization of the Oleogels Microstructure

**Figure 3:**
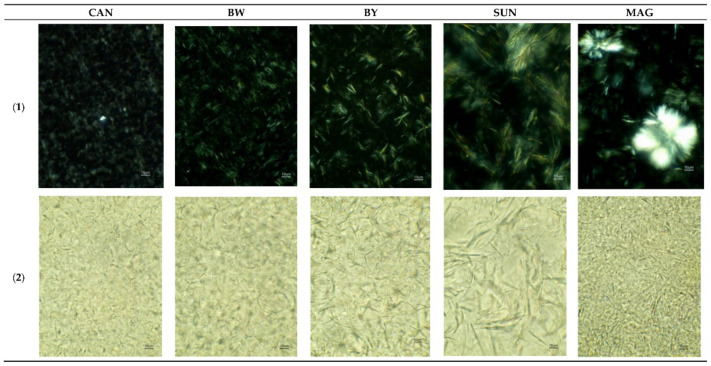
The microstructure of oleogels at 600× magnification, obtained by polarization (**images at the top-1**) and bright field (**images at the bottom-2**) for rapeseed oil oleogel structured with 5% sunflower wax (SUN), candelilla wax (CAN), bees wax white (BW), bees wax yellow (BY), or monoacylglycerols (MAG). Taken from Kupiec et al. [66]. Copyright 2020 Creative Common Attribution License.

Network formation during oleogelation can be observed with polarised microscopy due to the birefringence of fat crystals; however, the inconvenience is related to the micrometric scale limitations. Since waxes are one of the most efficient crystalline oleogelators, the in-depth morphology and the gelling mechanism has become well known after having been analyzed in numerous studies, and it is concluded that their gelling behavior is governed by the polarity of the solvents, minor oil components and oxidation reaction products, the wax crystals morphology and surface area, and the wax-solvents interactions [7,50,65]. For instance, rice bran wax in rice bran oil crystallizes as large dendritic crystals and the resulting network is loose and incapable of retaining the liquid oil inside it, but it also forms a fibrous morphology in other oils [42], while beeswax presented the same crystal arrangement and morphology when dispersed into medium chain triglycerides and long chain triglycerides [36]. In the study of rape seed oil oleogels, it was concluded that the more esters there are in the composition of a wax, the more needle-like a form (Figure 3) will result in the networks of structured oils; on the other hand, fat clusters irregularly distributed were observed for rape seed oil oleogel based on monoacylglycerols [66].

The morphology of the oleogels obtained using novel food grade oleogelators is of high interest and allows us to elucidate the mechanism of network formation. Microscopy was the perfect tool for assessing the performance of three types of sorghum waxes as new oleogelators for fish oil. The crystal shape of different sorghum waxes observed with polarized light microscopy were comparable to carnauba wax or candelilla wax [67]. External factors applied during oleogel formation (high cooling rates and ultrasonication) influenced the crystal size, which were smaller and denser [67]. This was also reported in the case of other waxes [50,65].

Sugarcane wax and its fractions formed oleogels of refined soybean oil, which were similar to other oleogels formed by candelilla, rice bran and carnauba wax [68]. The registered SEM images were not specific to wax-based oleogels, mostly open cell foams being visible, which formed a network due to chemical interactions (such as steric hindrance) that assures the oil binding capacity of sugarcane wax fractions [68]. For the crystal aggregates, which can be observed under polarized microscopy, a morphology such as fibre is considered ideal for gelation because of its higher surface area, than the sphere (specific to candelilla or beeswax) or a platelet morphology (developed by sunflower wax) and leads to stronger gels. This is because of more contact between the gelator molecules with each other or with the solvent.

γ-oryzanol and β-sitosterol self-assemble and form a fibrillar network, a gradual, structural transition being observed when the ratio between this gelator molecules or the purity of the compounds, vary [69]. Crystals grew further due to environmental conditions or concentration, and their appearance transformed from tubular into ‘spherulitic like’ or needle-like, as characterised after observation under polarized light microscopy [69]. Because phytosterol crystals are prone to migration during storage, causing oil loss, and monoglycerides (MGs) also lead to stability issues, to eliminate the inconveniences, they were combined in the formation of an oleogel of sunflower oil [39]. An improved network was seen under polarized light microscopy due to the separation of big MGs crystals and their disintegration caused by the mixed gelator mechanism, and plate-like crystals were also observed in the samples with higher phytosterol concentration [39]. Under electron microscopy, both gelators exhibited plate-like morpohology, but monoglycerides are formed by lamellar structures, which stack together and hold the liquid oil, while phytosterols improve the network’s oil binding capacity [38].

The formation of an oleogel structured with various concentrations of monoglycerides (7–12%) during cooling of the sample, was analyzed under polarized light microscopy and it was revealed that morphology of the MG was needle-like and the network formation occurred in three main steps: crystal nucleation, crystalline branching, and crystal growth [13,39,70]. However, the crystal morphology varies due to the oil’s composition and larger spherulitic or rosette-like structures were also formed [71].

Monoglyceride (MG) molecules are generally arranged as inverse lamellas in oil, with the hydrophobic tail connected to the vegetable oil and the glycerol head leading to an inverse orientation due to its hydrophilic nature, resulting in the formation of a two-dimensional close pack known as a hexagonal lattice, which entraps the liquid vegetable oil [72].

However, the high surface activity of this gelator might influence negatively on the macroscopic properties of the future oleogel-containing food products in terms of stability.

The morphology of ethyl cellulose oleogels was observed with cryo-SEM and AFM, and the oil droplets were separately positioned within an open polymer network, which presented pockets and pores interconnected by a network of capillary channels [73]. Their diameter was inversely proportional to the concentration of gelator and varied with different oil types or due to minor components such as oxidative by-products and polar molecules [14].

High oleic sunflower oil (HOSO) was recently structured with a ternary mixture consisting of different concentrations of ethyl cellulose, monoglycerides and candelilla wax as gelators, and under polarized microscopy only plate-like and granular crystals were observed, since ethyl cellulose do not form birefringent crystals upon cooling [74]. However, ethyl cellulose caused an impact on the microstructure of the oleogel and reduced the monoglyceride crystal occurrence [74].

Globular reverse micellar structures with an internal aqueous phase forming a 3D network due to their interactions were observed with light microscopy in the sunflower oil oleogel formed with Span-80, Tween 80, and water. Span-60 changed the crystal appearance of stearic acid soybean oil oleogels, from plate-like, to a branched structure with a “mesh-like” appearance; depending on the concentration of the ester, both plate-like crystals and fiber-shaped crystals were present in the observed micrographs, which revealed the synergistic effect of the gelators at a certain ratio [75].

From microscopy analysis and microstructures of an oleogel sample structured with a mixture of oleogelators, it can be concluded whether gel formation is based on the independent self-assembly and self-organization of each gelator, but the co-assembling between two or more gelators cannot be confirmed, XRD being more suitable in this case.

To analyze the network formation mechanism and phase transitions of the oleogel, rheology is commonly involved. The oleogel structure is confirmed when G′ > G″, high storage modulus (G′) in the oscillatory rheological measurements (amplitude sweeps) at low applied shear (τo) indicating a high gel strength [62].

The loss tangent is the ratio of the loss modulus G″ over the storage modulus G′ represents a measure of how elastic (tan δ < 1) or plastic (tan δ > 1) the oleogel can be at different temperatures.

Dynamic amplitude sweep oscillation tests are conducted to determine the resistance of the oleogel to deformation, to gather information about their elasticity, and to define the linear viscoelastic region (LVR). The LVR is a plateau formed during the increase in the oscillation stress, when the storage modulus (G′) and loss modulus (G″) values appear to be constant for a period of time [76].

The critical stress (τ*) is referred to as the terminal region of the LVR where the values of G′ differ with values different by 10% of the G′_LVR_ value [62].

The structure recovery ability of an oleogel increases the practical application and will assure that the oleogel is resistant to shearing, the thixotropic behavior being commonly monitored via the three-interval time test (3-ITT), the fraction of the recovery in the last interval being the studied parameter, which explains the viscosity changes in oleogel due to other factors [77]. So far, numerous oleogels showed a partial structure recovery [62,78,79]. When a material can easily recover its structure, it will be less susceptible to changes occurring during high shear conditions. In some studies, rheological measurements have been included to determine structure recovery, and the results show that these oleogels showed partial structure recovery upon shearing. This will be of high importance for production on industrial scale, if the oleogel transportation to different processing equipment will be realized through pipes [1].

The mechanical reversibility of ethyl cellulose-monoglyceride-candelilla wax oleogels was determined by Rodríguez-Hernández et al. [74], when the pre-sheared sample was cooled from 150 °C or 90 °C to 5 °C and an amplitude sweep from 0.004 to 100% and a frequency of 1 Hz. The viscoelastic properties of the oleogel were determined in static conditions at the zero moment and after 30 min and the oleogels’ recovery was determined [74].

The quality of an oleogel can be dictated by the oil binding capacity, which can be measured with centrifugal or dispersion analysis, the methods required for this analysis being critically reviewed elsewhere by Flöter et al. [9]

### 3.3. Chemical Characterization of the Oleogels

Gas chromatography is an analytical technique commonly used for the determination of the physicochemical properties of the oleogels, more precisely for the fatty acid composition of the resulting oleogels, the oils or lipidic gelators involved in the process, but is a very discriminatory technique and presents a broad range of application. Mostly it is conducted with the aim of assessing the decrease in the saturated or trans fatty acids of a food product, which is reformulated with oleogels, but also the effect of shear or temperature over the quality and integrity of the fatty acids or of the bioactive compounds [80]. The determination of the fatty acids is possible due to methylation and the comparison of their retention times with the standards. There are some inconveniences due to expensive columns and standards and the undesired oxidation or isomerization of the fatty acids which might occur. Park et al. [81] extracted the lipids from the oleogel using a modified version of the Folch method prior to their conversion into methyl esters and their analysis, while Gómez-Estaca et al. [26] applied derivatization directly on the freeze-dried oleogel containing a pâté sample. The degree of the pork backfat replacement with olive, linseed, a fish mixture of oils gelled with ethyl cellulose or beeswax dictated the fatty acid profile of the pâté, the saturated fatty acid proportion being decreased, and the polyunsaturated increased, while MUFA experienced fewer quantity variations [26]. An oleogel produced with 12% monoglyceride and amaranth oil presented a balanced fatty acid profile, and in comparison to cocoa butter, presented a lower stearic acid content (7.38–8.71%, as compared to 36.36%) and a higher unsaturated fatty acid content, but similar solid structures [70]. Higher amounts of palmitic acid were detected in an oleogel cream cheese in comparison to the oleogel and the constituent oil, and this is due to the contribution of milk fat-derived products added for the food product formulation [81]. By implying GC, Moghtadaei [82] concluded that the cooling temperature or the concentration of the structuring agent, namely beeswax, did not influence the fatty acid profiles of the oleogel sample, which was abundant in linoleic acid. High-intensity ultrasound application on a high oleic sunflower oil structured with myverol monoglicerides did not statistically affected the main composition [31].

To reveal the role of different chemical constituents of waxes which are commonly involved in oleogelation and to identify the chain length of the chemical components, the free fatty acids (FFA), and the fatty alcohols (FAL), Gas chromatography–mass spectrometry was applied on samples collected from the previous High Performance Liquid Chromatography separation with an evaporative light scattering detector (HPLC-ELSD), derivatization being necessary for the FFA and FAL because of their polar nature and non-volatility, which did not permit the movement in the column [35]. Palmitic acid was determined in each wax and it was the most abundant in beeswax, while candelilla wax also presented a particularity of being composed by longer alkyl chain free fatty acids. After saponification of the wax esters, it was concluded that they are comprised of a short-chain FA moiety bound to a longer-chain FAL moiety.

Moreover, gas chromatography coupled with a diode array detector (DAD) was applied by our group for evaluating the oxidative quality of oils and oleogels based on the quantification and identification of the volatile compounds.

### 3.4. Oxidative Stability

Since oleogelation requires high temperature treatments, the oxidative state of the oleogel can be affected, decreasing its quality. Proton nuclear magnetic resonance (pNMR) spectroscopy is a powerful technique for investigating the degradation of the polyunsaturated fatty acids and for the detection of the primary or secondary oxidation products, due to its capacity to allow structure elucidation even from complex lipidic mixtures. This is a non-invasive method based on assessing the chemical displacements values (shifts) expressed in ppm, defined as the absorption of electromagnetic radiation by protons, at an intensity different from that of the magnetic field applied by a device, to compensate for the shielding effect, offering protection due to the electric field induced by its own covalent electrons [83]. Peaks registered between 0–6.0 ppm are due to different functional groups of the triglycerides, one disadvantage of this method being the overlap of the peaks exhibited by some compounds.

Hwang et al. [84] observed the losses of olefinic and bis-allylic protons, along with losses of eicosapentaenoic acid (EPA) and docosahexaenoic acid (DHA) in oleogel samples evaluated by ^1^H-NMR and concluded that waxes have a prooxidant activity towards fish oil. Allylic compounds such as olefinic, allylic and bis-allylic compounds are primary oxidation products, which are easily transformed to secondary products, the rate of the oxidation process of unsaturated fatty acids being depicted by the bis-allylic carbons detected. In the work of Fu et al. [85], the relative changes of aliphatic to olefinic (R_ao_) and aliphatic to diallylmethylene (R_ad_) proton ratios during oxidation are calculated and also plotted against corresponding TOTOX values to assess the oxidative stability of EC oleogels.

Primary oxidation products of a conjugated nature were also determined by other authors [86] in soybean oil or peanut oil oleogels structured with a mixture of sitosterol and soy lecithin (4:1), along with epoxidation of the double bounds for soybean oil and changes in the functional groups in both kinds of oleogel samples, which were also enriched with acid or alkaline blueberry extracts (AC or AL) and resveratrol (R), of which AC and R might have an antioxidant effect. No secondary oxidation products were detected [86]. ^1^H-NMR spectra signals of primary oxidation products such as hydroperoxides and conjugated olefinics can be detected at δ = 5.0–9.0 ppm, while δ = 9.0–9.8 ppm are characteristic for the aldehyde proton signals and signals near δ = 8.20 ppm for hydroperoxides [86].

The protons of conjugated dienes and aldehydes resulted during the oxidation of ethyl cellulose oleogels were detected due to the peaks which appeared at 5.5–6.3 and 9.5–9.9 ppm on the spectra and during storage olefins. Diallylmethylenes were gradually reduced, while the aliphatic protons increased [85]. An important conclusion drawn by Fu et al. [85] is that the type of oil is of utmost importance in the oxidative stability of ethyl cellulose oleogels, since the fatty acids containing two double bonds degraded more rapidly than those with one double bond.

An innovative method to detect oxidation is the color penetration experiment, which was tested by evaluating the efficacy of wax-based oleogelation in preventing fish oil oxidation. This is based on the assumption that the rate of the diffusion of a dye can be correlated with the diffusion of oxygen through the sample, advanced color penetration being present in more oxidized samples [84]. Tests were also conducted on sunflower oil gelled with beeswax with 1:1 addition of ethyl alcohol with dissolved Sudan II dye and improved by the quantitative evaluation of the migrated dye by means of graduation chart [87].

A TBARS assay is also commonly used to evaluate the oxidative stability of oleogels or oleogel-containing food products, but is not as reliable since it can only detect malondialdehyde amounts and will not offer an entire picture of the real oxidative state or the presence of other primary or secondary degradation compounds. The sample preparation implies high temperature treatments in order to stabilize the content of the resulting malondialdehyde and, in this case, oxidation is rather induced and artifacts can be produced, other inconveniences being represented by the necessity of high purity reagents and, for some variations of the method, a centrifugation step to separate the colored resulting pigments. In a study which evaluates the oxidative stability of soybean oil structured with mixtures of cellulosic derivatives, samples are incubated in boiling water for two hours and 1-butanol is used instead of distilled water for the sample and reagent preparation [54]. The results indicated a higher rancidity for the samples containing Avicel, due to the carboxyl group originating from the carboxymethylcellulose from its composition [54]. Other authors dispersed the oleogel sample in 0.26% butylhydroxytoluene (BHT) by sonication, prior to the addition of TBARS reagent composed of 15% trichloroacetic acetic acid, 0.375% thiobarbituric acid and hidrocloric acid, and incubated it for only 15 min in boiling water followed by the addition of 4M ammonium sulphate and 4 hexane, and centrifugation [26]. Moreover, they also evaluated the oxidative state of a lipid pâté formulated with EC or BW oleogel and revealed that the oleogelation process due to temperature and air inclusion during shear, induced oxidation of lipidic compounds [26]. The highest oxidation values were detected for the ethyl cellulose oleogel containing pâté (partial replacement of pork fat) and during the storage experiment, a peak followed by a decline in the TBARS values after 30 days was registered.

For the evaluation of the oxidative stability of high oleic sunflower oil and oleogel designed for the incorporation in a cheese cream product, the addition of deionized water along with the methanol BHT solution and TBA solution and the separation by centrifugation of the pink aqueous portion for the spectrophotometric analysis was performed [81]. The method was not suitable for the evaluation of the oxidative status of the cream cheese product due to some possible interactions of MDA with the proteins, but it was revealed that the oleogelation process slightly negatively influences this property, because during storage no significant difference occurs between the oleogel and the bulk oil [81].

The analysis of the peroxide values indicates the presence of primary oxidation products and offers information about the oxidation state of an oleogel or food product, Codex Alimentarius (2001) indicating the maximal admitted values [16]. The method can be based on titrations with sodium thiosulfate (the effectiveness of which depends on the appearance of the oleogel) or on the oxidation of ferrous to ferric ions via peroxides and spectrophotometric determinations. Park et al. [81] reported an increase in the peroxide values both due to the heat implied in the oleogel formation and storage.

Rancimat tests are also performed in order to determine the oxidative state of oleogels by determining the longest induction time, which is the time from the beginning of the experiment to the starting point for oxidation [66].

Oxidation can be monitored by using a gas chromatographic method for the determination of propanal, a secondary oxidation product, during storage at 45 °C, a monoglyceride-based code oil oleogel developing less propanal in comparison to an algae oil oleogel [88].

### 3.5. Digestibility

The majority of the lipid digestion and absorption takes place in the stomach, where it seems that vegetable oil is extracted from the oleogel composition and can bind and deliver lipophilic bioactive molecules, such as β-carotene, the oleogel playing a vehicular role [89]. The function which oleogels can play as mechanisms of tailoring the digestibility of bioactive compounds is presented elsewhere [4]. It is already stated that the structure of the oleogel will influence the digestibility and bioaccessibility of some compounds, such as lycopene, co-enzyme Q10, DHA, EPA, conjugated linoleic acid, tannins, plant sterols, and isoflavones, which are fat soluble substances included in the oleogels composition for their delivery [90]. The type of structuring agent (low molecular weighted or polymeric) seems also to influence the lipolysis patterns, as revealed by [91].

*In vitro* digestion is mimicking all the steps traversed by the food from ingestion until adsorption. It starts from the mouth phase, where saliva fluid is simulated with 3% mucin and select minerals, and is followed by the stomach phase where pepsin is further added, temperature and Ph are properly adjusted, and stirring is performed and ends in the small intestine phase [69]. The last phase involves the incubation at 37 °C and the addition of the simulated intestinal fluid, bile salt solution, lipase solution and NaOH 0.1 mol/L, lipid digestion being evaluated based on the percentage of free fatty acids released [69].

Besides digestibility, it is important to know the effect of oleogels on human nutrition, as it is highlighted in the research conducted on coconut oil oleogel implication on the human blood triglycerides, glucose, insulin levels, and appetite [92].

## 4. The Macroscopic Scale of Oleogels

### 4.1. Critical Gelation

A compulsory test for evaluating whether an oleogel is formed or not is required to invert the recipient where the mixtures are placed to allow gel formation, for the visual observation of the content, the conclusion being based on whether the resulted structure is self-standing or presents flowing behavior [25,36,45,70]. Qualitative phase diagrams are often realized to describe the state of the resulting structures (gel, thick liquid, or liquid), as a function of the concentration of structuring agents and the temperatures to which oleogels are subjected [36]. The minimum concentration (% *w*/*v* or *w*/*w*) of a gelator involved in the formation of an oleogel with a specific oil, is defined as critical gelator concentration (CCG). In the case of mixed structuring agents, an empirical screening is conducted in order to determine the optimal ratio combinations [39,53].

When added to pre-heated oils in concentrations as low as 5%, the mixture of an alcohol and the correspondent fatty acid will produce relatively soft oleogels. A stearyl alcohol:stearic acid mixture (SO:SA) is the most studied system, but recently, behenyl alcohol and behenic acid have also proved structuring capacity [93].

Rice bran wax, beeswax, candelilla wax, sunflower wax, shellac wax, and carnauba wax are food grade low molecular weight gelators which have already demonstrated applicability in the oleogelation process as single or co-structurants and were successfully included in fat-rich food products with an improved nutritional profile or functionalities [94,95,96,97,98,99,100]. Waxes could provide excellent crystallization properties to liquid oils even at low concentrations due to their low polarity, long chain length, and relatively high melting point. However, the wax chemical constituents (alkyl ester chain length, degree of saturation, minor components or impurities) dictate the appearance of ordered primary crystals, their morphology, and the resulting networks’ gelation capabilities [101]. The critical gelling concentrations of waxes is influenced by the polarity of the solvents [65], which can be described by analyzing the dielectric constant and the viscosity of the oils [71].

Sorbitan esters (SPAN 40, 60, and SPAN 65) are low molecular weight gelators which have demonstrated structuring capacity; different concentrations (ranging between 15–20%) are needed in order to gel some edible oils for drug delivery purposes [55,64,102]. For instance, 17% Span 60 added to mustard oil will transform the system into an oleogel [55]. Surfactants such as Tween 20 and Tween 80 are frequently used as co-gelators since better entrapment, solubility, and drug permeation was observed [64]. A fluid-filled structure technique was used to elaborate oleogels with a 2:1 ratio (*w*/*w*) of Tween 80 and Span 80 but with up to 80% water addition [103].

Oleogels developed using mono-component phytosterols are suitable only for some food products, because the water from food matrix bonds to the hydroxyl group of phytosterols leads to the formation of hydrate crystals, which break up the network of oleogel [104]. β-sitosterols and γ-oryzanol mixtures are commonly used to impart structure to various oils, of which minor components, viscosity or polarity, influence the firmness of the oleogel and the packing of the tubular structures [15]. Solid-like gel was developed by the addition of water to an equimolar mixture of lecithin and oryzanol due to the formation of some miscellaneous structures and hydrogen bonding, as a result of the interaction of the phosphate group belonging to the lecithin and the phenol group of the oryzanol [105]. Monoglycerides and phytosterols are also interacting in the oleogelation process and form a mixed crystal system. It was stated that sterol-based compounds are stopping the polymorphic transitions of monoglyceride in a complex system [39]. Native phytosterols are esterified with fatty acids to lower their melting point or are co-crystallised with other components, such as monoacylglycerols and glycerin monostearate, the latest systems also presenting whippability and forming oleofoams, depending on the cooling rates and the concentration of the gelators [106].

### 4.2. Solid Fat Content

The analysis of the content of solid fat in oils and fats is important for bakery products, sweets and margarines, where the type of fat plays a basic structural and technological role, affecting also the appearance and properties of organoleptic, spreadability or plasticity. The traditional method is dilatometry, but the analysis of the solid fat content by nuclear magnetic resonance is also standardized (AOCS Cd 16b-93; ISO 8292-1 and IUPAC 2.150). There are two ways to measure this parameter: by a direct or an indirect method. The method directly measures the signal from both the liquid and solid components of fats, being a simple and fast method, while the indirect method compares the signal of the liquid components and compares them with the signal of the completely melted sample. The possibility of determining SFC by velocimetry (ultrasound reflection measurement) was also proposed, but it was concluded that this method it is not suitable for any type of fat system. Fats contain both finely dispersed and interconnected solid fat crystals of Van der Waals forces and liquid fat at room temperature, the plasticity resulting from the dominance of the solid component over the liquid one.

### 4.3. Texture and Appearance

It is already stated that the use of oleogel as a trans or saturated fat replacer in foods has implications on food texture. Mechanical properties of the oleogels are described by the firmness or hardness of the system. Firmness is defined as the force required to create a given deformation under given conditions and is reported in grams or newtons, and stickiness is defined as the force needed to pull out or remove the probe or knife from the sample. The firmness of the oleogel is usually correlated to the type of network observed with microscopy, high values indicating a denser network.

The texture profile analysis (TPA test) is represented by a two cycle penetration of the sample with a cylindrical probe commonly conducted [107]. Firmness, adhesion, spreadability, chewability, and gumminess are the main textural parameters analyzed with a texture analyzer. Oleogel texture can be determined according to the back extrusion methodology, from force-time curves maximum force (peak force during back extrusion) and tackiness (adhesive force peak during probe return after reaching the programmed depth) being calculated [54]. The spreadability of a citrus fiber-based oleogel was determined by a compression test using the TTC spreadability rig (consisting of a cone probe which precisely matched the cone-shaped product holder) in comparison to a cocoa-hazelnut spread, the information being gathered by measuring the firmness and the work of penetration, which was the work calculated to push the cone probe down 45 mm into the sample [108]. It was concluded that the type of oil (liquid palm oil or olive oil) did not affect the spreadability and similar values were obtained for the less shear-activated citrus pectin and the reference [108]. Extrudability and spreadability of olive oil and soybean oil oleogels structured with Span 40 and Span 40/Tween 80 were studied with other method which measured the distance travelled by the oleogel extruded from two glass slides of equal weight [64].

In oleogels structured with stearyl acohol and stearic acid, a higher firmness were registered in the back extrusion tests for the mixture with the ratio in the favour of stearyl alcohol [30]. Higher firmness was registered in the penetration test for the oleogel prepared with the 1:4 mixture of β-sitosterol and stearic acid compared to the oleogel structured with only stearic acid or other combinations, revealing that sitosterol influences the packing of the stearic acid due to the fibril network, which fills the space in the network of SA crystals, transforming it into a dense structure [45]. The extent to which oleogels are similar to the type of fat they aim to replace in food ingredients, from a textural point of view, is discussed elsewhere [109].

The colorimetric analysis aims to characterize the color of the oleogels in a fresh state or during storage by reflectance measurements, based on the opacity or brightness parameters (L*), red and green chromatics (a*) and blue and yellow chromatics (b*), the CIE-L*a*b* uniform color space (CIE-Lab) being involved in recording the color. Additionally, Hue angle (h*) and Chroma (C*) can be calculated [110]. It also compares the color of the oleogel with the matrix of the food in which it is used, because this parameter is important in sensory analysis and in the acceptance of innovative products by consumers. High values of transparency for the oleogel are desired because in this manner it will not influence too much the appearance of the food products in which they are incorporated.

### 4.4. Sensorial Analysis

The food products developed with oleogels as ingredients are subjected to sensory analysis in order to evaluate the consumer acceptance or some aspects which might not be detected by analytical methods. Even if the results of research indicate that oleogels are capable of replacing some trans or saturated fats in food products, the consumer acceptance will dictate the final formulation composition [109]. Hedonic tests are describing acceptance scores for attributes such as color, flavor, texture, aroma, overall appearance and impression, and even spreadability [111].

Oleogels based on 8% Beeswax linseed oil or oleogels designed with a 60:40 (*w*/*w*) mixture of γ-oryzanol and β-sitosterol were used to partially replace pork backfat in fermented sausages, and the products containing 20% oleogels in the formulation were scored in the positive part of the hedonic scale along with the control batch, while higher levels of substitution were found unacceptable and the oleogelator type was not found to influence overall acceptability. This was not the case for pâtés, where ethyl cellulose oleogels caused decreases in the color, taste, and overall acceptability [26]. Curcumin addition to pork burgers formulated with beeswax oleogel reduced the sensory acceptability, and thus was not recommended to be used in this formulation for the anti-oxidant effect which it demonstrated [112].

A check-all-that-apply (CATA) questionnaire with 23 terms related to the sensory characteristics along with a hedonic test was evaluating the acceptance of Bologna sausages with oleogel made with pork skin, water, and high oleic sunflower oil (HOSO) (1.5:1.5:1) and a substitution of up to 50% was found to be acceptable [113].

Sensory evaluation of emulsion oleogel containing cake slices was performed in controlled illumination and temperature rooms by a 5-point hedonic scale, for evaluating the texture, color, taste, and overall preference, the results indicating that substituting the shortening with oleogels up to 75% was found acceptable for the cakes’ organoleptic properties among the sensory evaluators [114]. Butter was replaced in cakes with tea polyphenol-palmitate and citrus pectin camellia-oil oleogels and the 1.5% and 2.5% citrus pectin oleogels can be labelled as sensory acceptable after the 7 points hedonic scale sensory analysis of the crust color, crumb color, odour, flavor, and texture [115].

## 5. Conclusions

XRD analysis is characterizing the building units of the oleogel network on a nanometre scale and it can be used to determine the crystallization temperature required for the formation of a well-ordered structure and the co-crystallization of multiple structuring agents or the polymorphic transitions. The main inconvenience regarding this method being that is suitable only for the crystalline structures. Both lipidic and non-lipidic structuring agents can be visualised with different microscopy techniques, which allow the visualization starting from a nanometre scale, but imply the preparation of the sample prior to visualization and might deteriorate the original network. Furthermore, the study of the molecular interactions which are the base of the network formation in the oleogel can be studied with Fourier Transformation Infrared Spectroscopy (FTIR) and Raman Spectroscopy. The main disadvantage is that mixed signals are registered due to the complex chemical composition and similar constituents which lead to the formation of overlapped signals. Aggregation of the crystalline structures can be detected with Dynamic light scattering or laser diffraction, which were less applied in the recent research regarding edible oleogels.

Differential Scanning Calorimetry (DSC) is one of the methods describing the oleogel on a micrometre scale and it determines the melting and crystallization points of the oleogels but they are not thermally characterising the formation of the oleogels since no shear of the sample is involved during the analysis. Meting temperatures could also be determined by the falling ball method. Moreover, the crystallization induction time can be evaluated with a spectrophotometer or rheologically, along with the network formation, mechanical properties or response towards external forces. Accelerated stability studies consist of thermo-cycling or freeze–thaw cycling, along with syneresis measurements conducted in order to assess the change in gelation time and to characterize the stability of oleogel during different manipulation steps.

So far, the chemical characterization of the oleogels is resumed to the determination of the constituent fatty acids and bioactive components, the volatile compounds release, the oxidation state, and the oil binding capacity. Visually, the formation of the oleogel can be determined and on a macroscopic scale and the critical gelation concentration parameter characterizes the oleogel formation. The functionality of an oleogel for the edible application is based on the textural analysis and the solid fat content, but colorimetry and sensory analyses also dictate whether an oleogel will or will not be accepted by the consumer, regardless of its capacity to mimic the properties of the conventional fats. The digestibility of the oleogels was also mimicked and the results are promising since it was revealed that they are useful for the delivery of bioactive compounds. Moreover, the impact of the oleogel consumption along with carbohydrates on the triglycerides, glucose, insulin levels, and appetite of the consumers were also addressed.

## Data Availability

Not applicable.
